# Complement factor D haplodeficiency is associated with a reduced complement activation speed and diminished bacterial killing

**DOI:** 10.1002/cti2.1256

**Published:** 2021-04-03

**Authors:** Jeroen D Langereis, Renate G van der Molen, Corrie de Kat Angelino, Stefanie S Henriet, Marien I de Jonge, Irma Joosten, Annet Simons, Janneke HM Schuurs‐Hoeijmakers, Marcel van Deuren, Koen van Aerde, Michiel van der Flier

**Affiliations:** ^1^ Department of Laboratory Medicine Laboratory of Medical Immunology Radboud Institute for Molecular Life Sciences Radboudumc Nijmegen The Netherlands; ^2^ Radboud Center for Infectious Diseases Radboudumc Nijmegen The Netherlands; ^3^ Pediatric Infectious Diseases and Immunology Amalia Children's Hospital Nijmegen The Netherlands; ^4^ Expertise Center for Immunodeficiency and Autoinflammation (REIA) Radboudumc Nijmegen The Netherlands; ^5^ Department of Human Genetics Radboudumc Nijmegen The Netherlands; ^6^ Department of Internal Medicine Division of Infectious Diseases Radboudumc Nijmegen The Netherlands; ^7^ Present address: Pediatric Infectious Diseases and Immunology Wilhelmina Children’s Hospital UMC Utrecht Utrecht The Netherlands

**Keywords:** complement, factor D, haplodeficiency, immunodeficiency, infection, vaccination

## Abstract

**Objectives:**

Complete deficiency of alternative pathway (AP) complement factors, explained by homozygous mutations, is a well‐known risk factor for invasive bacterial infections; however, this is less obvious for heterozygous mutations. We describe two siblings with a heterozygous NM_001928.3(CFD):c.125C>A p.(Ser42*) mutation in the complement factor D (fD) gene having a history of recurrent bacterial infections. We determined the effect of heterozygous fD deficiency on AP complement activity.

**Methods:**

We determined the effect of fD levels on complement activation as measured by AP activity, complement C3 binding to the bacterial surface of *Neisseria meningitidis* (Nm), *Streptococcus pneumoniae* (Sp) and non‐typeable *Haemophilus influenzae* (NTHi), and complement‐mediated killing of Nm and NTHi. In addition, we measured the effect of vaccination of complement C3 binding to the bacterial surface and killing of Nm.

**Results:**

Reconstitution of fD‐deficient serum with fD increased AP activity in a dose‐ and time‐dependent way. Reconstitution of patient serum with fD to normal levels increased complement C3 binding to Sp, Nm and NTHi, as well as complement‐mediated killing of Nm and NTHi. Vaccination increased complement C3 binding and resulted in complete killing of Nm without fD reconstitution.

**Conclusion:**

We conclude that low fD serum levels (< 0.5 μg mL^−1^) lead to a reduced speed of complement activation, which results in diminished bacterial killing, consistent with recurrent bacterial infections observed in our index patients. Specific antibodies induced by vaccination are able to overcome the diminished bacterial killing capacity in patients with low fD levels.

## Graphical abstract

In this study, we show that low complement factor D levels (< 0.5 µg mL^−1^) result in slower complement activation and increased susceptibility for infections by fast proliferating bacteria such as *Neisseria meningitidis, Haemophilus influenzae* and *Streptococcus pneumoniae*. Vaccination was able to overcome the slower complement pathway activity through robust initiation of the classical pathway.

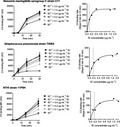

## Introduction

One of the first defence barriers after bloodstream invasion of bacteria is complement activation, leading to the covalent fixation of opsonising C3b molecules on the bacterial surface and, in case of Gram‐negative bacteria, to killing by the insertion of the membrane attack complexes (MAC) in the bacterial outer membrane.^1^ However, these processes will only be protective when they start in time and when they run fast enough to keep the exponential outgrowth of bacteria in control. While the lectin pathway (LP) and the classical pathway (CP) direct the site of complement activation to the bacterial surface, the alternative pathway (AP), sometimes called ‘Amplification Pathway’, is designed to accelerate the process of complement activation by generation of C3b that is both a product of AP‐convertase activity and the starting point for new and more AP‐convertase complexes. Absent AP activation, as occurs for instance in patients with Properdin or factor D (fD) deficiency, is associated with invasive meningococcal infections with a rapid and overwhelming outgrowth of bacteria in the bloodstream.^2^, ^3^, ^4^, ^5^, ^6^, ^7^, ^8^, ^9^ Apparently, complement activation in these conditions is too slow to control the growth of the bacteria.

Complete fD deficiency is a rare but well‐recognised cause of a defective AP complement activation that predisposes to overwhelming bacterial infections. Up till now, heterozygous mutations in the fD gene were not regarded as clinically important. In the present study, we present two siblings with low serum fD concentrations explained by a heterozygous NM_001928.3(CFD):c.125C>A p.(Ser42*) mutation. By measuring the AP activity, C3b fixation and bactericidal activity in the sera with low fD concentrations and in fD‐deficient serum supplemented with various concentrations of purified fD, we provide evidence that low fD concentrations (< 0.5 µg mL^−1^) results in a slower complement activation speed and bactericidal activity. These findings point towards novel type of immunodeficiency, that is characterised by an intact but too slow complement response, which predisposes to infections with rapidly proliferating bacteria that demand a rapidly accelerating response of complement.

## Results

### Patient details

Patient A was referred to our hospital at the age of 11 because of recurrent infections. His medical history revealed recurrent acute otitis media in the first 2 years of life. At 2 years of age, he suffered from a *Staphylococcus aureus* septic coxarthritis with bacteraemia, requiring intravenous flucloxacillin, with full recovery. At ages 2 and 9, he was diagnosed with bacterial pneumonia and received out‐patient antibiotic treatment. At the age of 11, he developed mastoiditis caused by *Streptococcus pyogenes* treated with intravenous ceftriaxone. He received all the childhood vaccines, including 10‐valent conjugate pneumococcal vaccine, *Haemophilus influenzae* type B and group C Meningococcus vaccine.

Immunological evaluation showed normal immunoglobulin levels and a normal response to pneumococcal polysaccharide vaccination. Evaluation of his complement status demonstrated a decreased AP of 50% (reference 67–133%), a normal CP of 114% (reference 67–149%) and a decreased serum concentration of fD 0.26 µg mL^−1^ (reference 0.96–2.13 µg mL^−1^). Analysis of 34 complement proteins in serum by mass spectrometry revealed undetectable fD and significant lower C1 inhibitor, C3, C5 and vitronectin serum level.^10^ Genetic analysis of complement genes revealed a maternal heterozygous NM_001928.3(CFD):c.125C>A p.(Ser42*) gene mutation. Additional analysis of 386 primary immunodeficiency genes using clinical exome sequencing was further normal. Following diagnosis, he was started on co‐trimoxazole prophylaxis and received additional meningococcal ACYW and 4C‐MenB vaccines.

Patient B was the only sister of patient A and was also diagnosed with complement fD deficiency (Heterozygous NM_001928.3(CFD):c.125C>A p.(Ser42*) mutation in the fD gene) at the age of 6. Until then, she suffered from recurrent acute otitis media and sinusitis, but had no history of severe bacterial infections. Complement evaluation demonstrated a decreased AP of 53% (reference 67–133%), a normal CP of 89% (reference 67–149%) and a decreased serum fD level of 0.30 µg mL^−1^ (reference 0.96–2.13 µg mL^−1^). Analysis of 34 complement proteins in serum by mass spectrometry revealed undetectable fD.^10^ Genetic analysis of complement genes demonstrated the familial heterozygous fD mutation. Following diagnosis, she was started on co‐trimoxazole prophylaxis and received additional pneumococcal, meningococcal ACYW and 4CMenB vaccines.

### Complement factor D haplodeficiency results in decreased complement binding and complement‐mediated killing

In order to determine the functional consequences of the lowered fD serum levels in our two patients, we reconstituted patient serum with purified fD up to 2.0 µg mL^−1^ and determined complement activity and complement‐mediated killing of Gram‐positive *Streptococcus pneumoniae* (Sp) and Gram‐negative *Neisseria meningitidis* (Nm) and non‐typeable *H. influenzae* (NTHi) bacteria.

**Figure 1:**
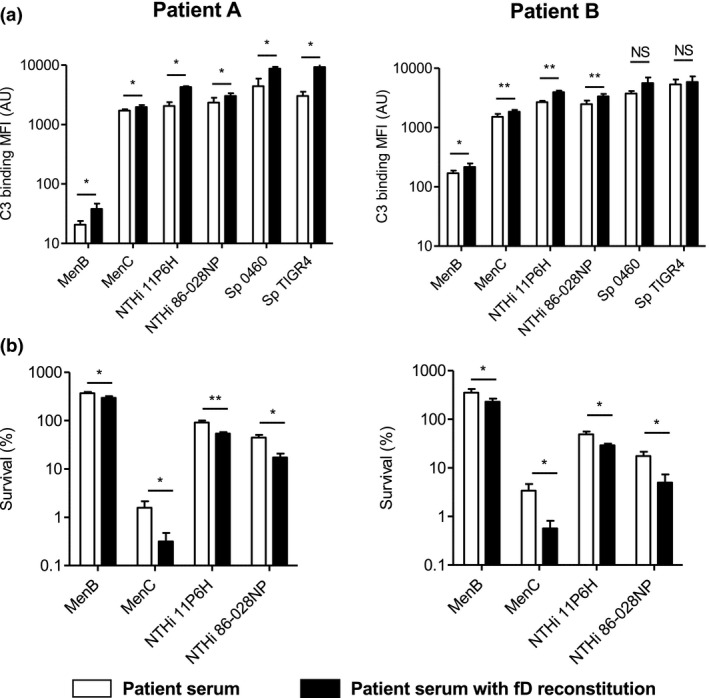
Reconstitution of patient serum with purified factor D increases complement C3 binding to the bacterial surface and complement‐mediated killing. **(a)** *N. meningitidis* serogroup B strain H44/76 (MenB) (*n* = 6), *N. meningitidis* serogroup C strain C11 (MenC) (*n* = 6), *S. pneumoniae* strain PBCN0460 (Sp 0460) (*n* = 4), *S. pneumoniae* strain TIGR4 (Sp TIGR4) (*n* = 4), NTHi strain 11P6H (*n* = 4) and NTHi strain 86‐028NP (*n* = 4) were incubated with 10% patient serum in HBSS3+ with and without factor D reconstitution for 30 min at 37°C, and binding of C3 to the bacterial surface was determined by flow cytometry. **(b)** *N. meningitidis* serogroup B strain H44/76 (MenB) (*n* = 6), *N. meningitidis* serogroup C strain C11 (MenC) (*n* = 6), NTHi strain 11P6H (*n* = 4) and NTHi strain 86‐028NP (*n* = 4) were incubated with 20% (*N. meningitidis*) or 5% (NTHi) patient serum with and without factor D reconstitution for 60 min at 37°C, and the number of bacteria was determined by colony‐forming unit (CFU) counts. A two‐tailed paired *t*‐test was performed to determine statistical significance. NS, not significant; *P‐value < 0.05; **P‐value < 0.01.

Reconstitution of patient serum with fD increased binding of C3 to the bacterial surface of Nm, Sp and NTHi (Figure 1a). Consistent with increased C3 binding to the bacterial surface as read‐out for complement activation, reconstitution of patient serum with fD increased direct complement‐mediated killing of Nm and NTHi (Figure 1b).

### *Neisseria meningitidis* vaccination compensates for decreased complement activity in patient serum

**Figure 2:**
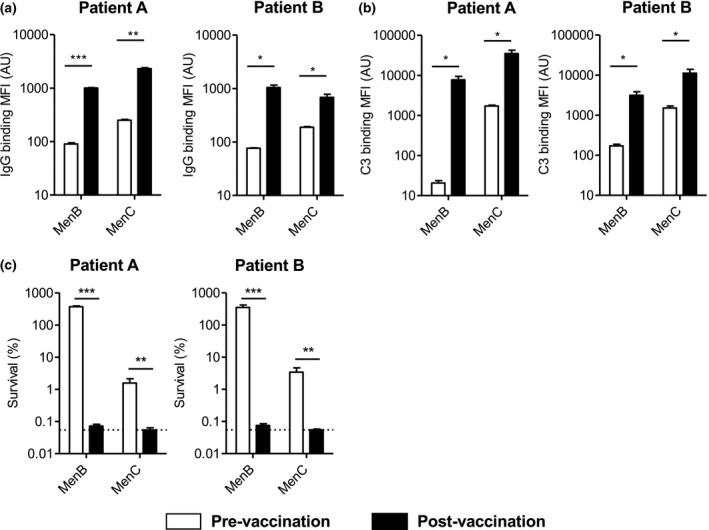
Vaccination increases complement C3 binding to the bacterial surface and killing of *N. meningitidis*. **(a)** *N. meningitidis* serogroup B strain H44/76 (MenB) (*n* = 6) and *N. meningitidis* serogroup C strain C11 (MenC) (*n* = 3) were incubated with patient pre‐ and post‐vaccination serum in HBSS3+ for 30 min at 37°C, and binding of IgG to the bacterial surface was determined by flow cytometry. **(b)** *N. meningitidis* serogroup B strain H44/76 (MenB) (*n* = 6) and *N. meningitidis* serogroup C strain C11 (MenC) (*n* = 6) were incubated with patient pre‐ and post‐vaccination serum in HBSS3+ for 30 min at 37°C, and binding of C3 to the bacterial surface was determined by flow cytometry. **(c)** *N. meningitidis* serogroup B strain H44/76 (MenB) (*n* = 6) (20% serum in HBSS3+) and *N. meningitidis* serogroup C strain C11 (MenC) (*n* = 6) were incubated with 20% (MenB) or 5% (MenC) patient pre‐ and post‐vaccination serum in HBSS3+ with and without factor D reconstitution for 60 min at 37°C, and the number of bacteria was determined by colony‐forming unit (CFU) counts. The horizontal dotted line represents the lower limit of detection. A two‐tailed paired *t*‐test was performed to determine statistical significance. NS, not significant; *P‐value < 0.05; **P‐value < 0.01; ***P‐value < 0.001.

We determined the effect of meningococcal ACYW and 4CMenB vaccination on complement activity and complement‐mediated killing of Nm strains using serum of both patient A and B sampled before and 5 months post‐vaccination. As expected, following vaccination, binding of IgG to the bacterial surface of Nm with post‐vaccination serum was significantly higher than that with pre‐vaccination serum (Figure 2a). Binding of C3 to the bacterial surface of Nm with post‐vaccination serum was significantly higher than that with pre‐vaccination serum (Figure 2b) for both patients. In addition, vaccination resulted in almost complete killing of Nm strains (Figure 2c).

### Complement factor D increases complement activation speed in a dose‐dependent way

**Figure 3:**
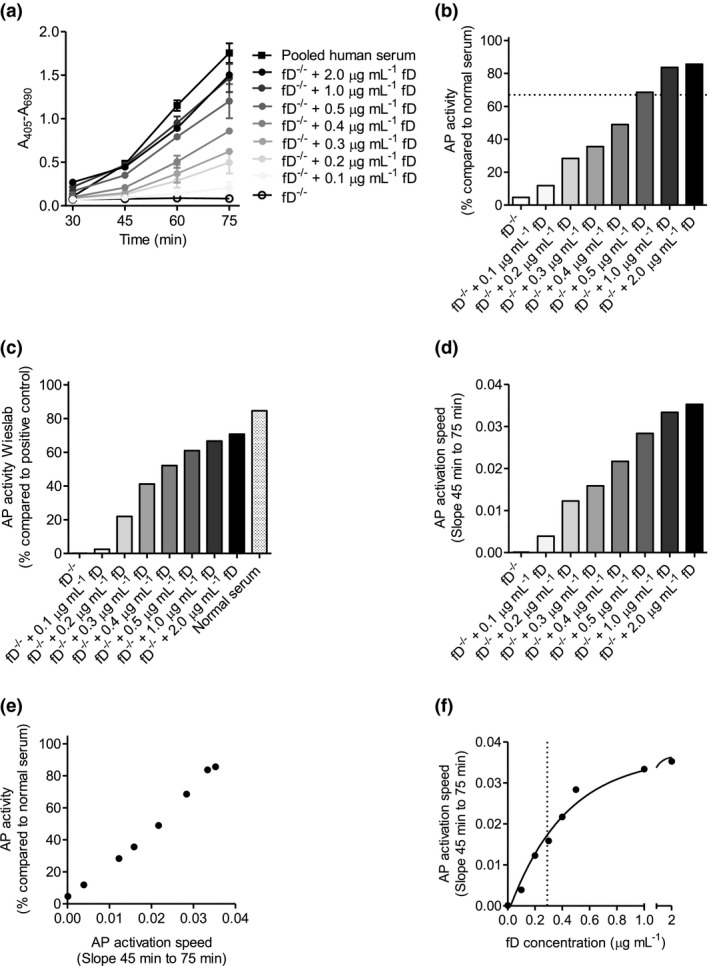
Reconstitution of factor D‐deficient serum with purified factor D increases complement activation speed in a dose‐dependent way. Factor D‐deficient serum was reconstituted with purified factor D, and **(a)** C5b‐9 formation was determined by ELISA after 30, 45, 60 and 75 min (*n* = 3). **(b)** AP was calculated at 75 min (the horizontal dotted line visualises AP of 67%), **(d)** and AP activation speed as determined by the slope between 45 and 75 min. **(c)** Factor D‐deficient serum was reconstituted with purified factor D, and AP activity was determined by Wieslab ELISA. **(e)** Linear regression between AP and AP activation speed was calculated. **(f)** One‐phase decay non‐linear fit between AP activation speed and serum factor D concentration. The vertical dotted line visualises the EC50.

To determine the effect of fD serum level on the speed of complement activation, we reconstituted serum from a fD‐deficient patient with purified fD. In order to capture complement activation speed, we determined the formation of the C5b‐9 complex after 30, 45, 60 and 75 min of incubation with fD‐deficient serum, fD reconstituted serum or pooled human serum as control. The CP was not affected by the absence of fD, nor by fD reconstitution (data not shown). In contrast, no activation of the AP was observed using fD‐deficient serum, and reconstitution of fD dose‐dependently increased AP activation (Figure 3a), reaching normal levels (AP > 67%, dotted horizontal line) with 0.5, 1.0 and 2.0 µg mL^−1^ purified fD, and a maximum activity with 2.0 µg mL^−1^ purified fD of 86% compared to normal serum (Figure 3b). Wieslab AP ELISA also showed no activity for fD deficient serum, and reconstitution of fD increased AP activation in a dose‐dependent manner (Figure 3c). However, the maximum AP activity with 2.0 µg mL^−1^ purified fD in the Wieslab ELISA was lower than (71%) the maximum AP activity in our AP activity assay (86%). The difference in maximum AP activity with 2.0 µg mL^−1^ purified fD is largely due to a higher complement activity of the positive control compared to normal serum, which shows 85% activity compared to the positive control in the Wieslab AP ELISA.

The speed of AP activation, as measured by the slope between time‐points 45 and 75 min, also increased in a fD dose‐dependent way (Figure 3c). Of note, the speed of AP activation was highly correlated (*R* ^2^ = 0.9923, *P*‐value < 0.0001) with the AP percentage as measured after 75 min (Figure 3d), indicating that the AP percentage is a reliable tool to estimate the speed of AP complement activation. The half maximal effective concentration (EC50) of fD on the speed of AP activation was 0.24 µg mL^−1^ (*R* ^2^ = 0.9933) (Figure 3e), which is consistent with an AP of ~50% for Patient A and Patient B having ~0.3 µg mL^−1^ fD.

### Complement factor D increases complement C3 deposition on the bacterial surface in dose‐dependent way

**Figure 4:**
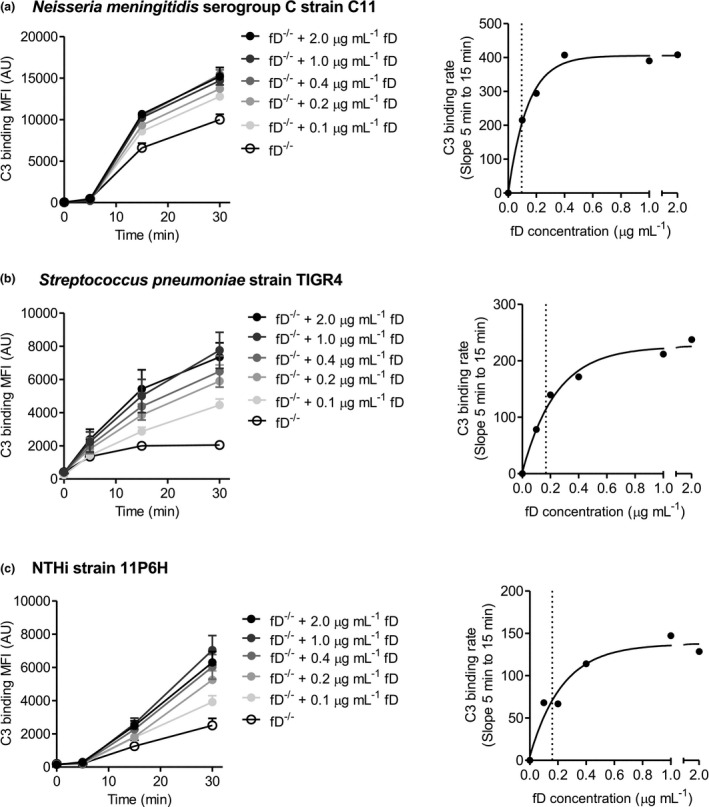
Reconstitution of factor D‐deficient serum with purified factor D increases complement deposition on the bacterial surface in a time‐ and dose‐dependent way. **(a)** *N*. *meningitidis* serogroup C strain C11 (*n* = 4), **(b)** *S. pneumoniae* strain TIGR4 (*n* = 4) and **(c)** NTHi strain 11P6H (*n* = 3) were incubated with 10% factor D‐deficient serum in HBSS3+ with or without reconstitution of purified factor D for 5, 15 and 30 min at 37°C, and binding of C3 to the bacterial surface was determined by flow cytometry. One‐phase decay non‐linear fit between C3 binding rate and serum factor D concentration was calculated. The vertical dotted line visualises the EC50.

Next, we determined the effect of reconstituting fD‐deficient serum with purified fD on complement deposition on the bacterial surface of bacterial pathogens Nm, Sp and NTHi. Binding of complement C3 to the bacterial surface of Nm, Sp and NTHi was time‐ and fD‐concentration dependent (Figure 4). The speed of complement activation, as measured by the increase in slope between time‐points 5 and 15 min compared to no fD, increased in a fD dose‐dependent manner with an EC50 of 0.09, 0.17 and 0.16 µg mL^−1^ fD for Nm, Sp and NTHi, respectively. These results show that the level of fD, and thereby the speed of complement activation, has a major effect on the clearance of rapidly proliferating bacteria.

## Discussion

Here, we demonstrate that a heterozygous NM_001928.3(CFD):c.125C>A p.(Ser42*) mutation in the fD gene can result in lower serum fD levels (**~**0.3 µg mL^−1^), evolving in a slower speed of AP complement activation and thus in a higher chance to acquire bacterial infections.

factor D deficiency is a rare condition with only six previously published case reports. The first publication of Kluin‐Nelemans from 1984 described a partial fD deficiency in a 40‐year‐old monozygous twin with upper and lower respiratory tract infections caused by *H. influenzae*, *Proteus mirabilis* and *Pseudomonas aeruginosa* since childhood.^2^ In 1989, Hiemstra *et al*. described a young man with complete fD deficiency, who had a *N. meningitidis* meningitis at the age of 14 and episodes of invasive *Neisseria gonorrhoeae* disease at the age of 19 and 24.^3^ Complete deficiency was also reported by Weiss *et al*. in 1998, in a 6‐day‐old baby with *S. pneumoniae* meningitis and sepsis.^4^ The first genetically confirmed case series dates from 2001,^5^ which described five family members of a highly consanguineous Dutch family with complete fD deficiency with the same, but in this case homozygous mutation as found in our study (NM_001928.3(CFD):c.125C>A p.(Ser42*)). Two of these fD‐deficient individuals suffered from invasive meningococcal disease at the age of 23 and 20, the latter died at the age of 71 from a fatal *S. pneumoniae* pneumonia and meningitis. In 2006, our group reported on two Turkish babies, a girl of 9 months and her brother of 13 months old, both with severe invasive meningococcal disease and complete fD deficiency due to a double homozygous mutation.^6^ At last in 2018, Sng *et al*. reported invasive meningococcal disease in a 19‐year‐old South Asian female with a type III fD deficiency; her younger brother had the same mutation but no disease.^7^ From these studies, describing 11 cases of complete fD deficiency, it can be concluded that fD deficiency may cause disease at any age. However, demonstrated by the identification of four affected healthy sibs with complete fD deficiency, the defect by itself does not influence normal childhood development, provided there is no contact with a virulent bacterial strains.

A second conclusion that can be drawn is that incomplete fD deficiency present in several family members carrying the heterozygous mutation does not predispose to increased susceptibility for infections. In three studies,^3^, ^5^, ^6^ 24 family members were identified with decreased, but not absent fD levels; none had a history of infections. However, fD concentration in most carriers was rather high; Biesma measured in 19 carriers a mean of 0.92 ± 0.24 µg mL^−1^,^5^ while fD levels in the family members without the mutation was 2.22 ± 0.68 µg mL^−1^, which was higher in comparison to 60 non‐related healthy donors (1.39 ± 0.32 µg mL^−1^). Of note, the fD‐gene mutation in the Biesma study was similar to the mutation as found in our study. Therefore, it appears that because of interindividual variability, some individuals with a heterozygous mutation will demonstrate lower complement fD serum levels, while others with the same mutation will have normal complement fD serum levels. Kluin‐Nelemans, the first publication on partial fD deficiency, reported a lowered fD level of 8%, that is between 0.08 and 0.17 µg mL^−1^ with a normal range of 0.96–2.13 µg mL^−1^.^2^ The two patients in this study showed a remarkably similar clinical picture with recurrent infections as compared to our two patients.

Recently, we have determined serum levels of 34 complement proteins in 40 healthy controls, 83 patients with a diagnosed deficiency and 14 individuals with a potential pathogenic variant. Among the patients, three patients with a heterozygous NM_001928.4(CFD):c.744C>G p.(Ile248Met) mutation were included. For all three patients, a lower fD serum level was found, but no clinical presentation or history of these patients was available.

Despite the fact that both of our patients were carriers of the same mutation in fD resulting in a substantial lowered serum fD level, patient A experienced multiple invasive infections, whereas patient B did not. We think that this is largely due to the chance that an individual encounters a virulent bacterial strain. For instance, the Biesma study describes three family members with a complete factor D deficiency who did not have a history of recurrent infections,^5^ and similar reports have been published on patient with other complement deficiencies including C5.^11^ Other differences in the complement pathway proteins could also contribute to a difference in disease susceptibility. For patient A, significant lower levels of C1 inhibitor, C3, C5 and vitronectin were detected.^10^ In addition, and especially relevant for complement deficiencies that can be compensated by sufficient CP activation, nasopharyngeal colonisation or subclinical infections induce immunisation that would protect against reinfection.

Besides genetic variations resulting in lowered serum factor D levels, differences in adipose tissue have been shown to affect serum factor D levels. Unlike most complement proteins, which are mainly produced by the liver, factor D is largely produced by adipose tissue.^12^, ^13^ As a result, patients with congenital generalised lipodystrophy have a lowered serum factor D level.^14^ Wu *et al*. also showed factor D titration experiments on AP activity by haemolytic and microtitre plate assays. In both assays, a factor D concentration‐ and time‐dependent increase in AP activity was observed.^14^

We show that fD plays a pivotal role in the AP complement activation, which has also been acknowledged by the pharmaceutical industry who have multiple fD blocking or inhibiting drugs in their pipelines for diseases that are characterised by an over‐active or uncontrolled complement system. For example, patients with paroxysmal nocturnal haemoglobinuria lack the presence of complement‐inhibitory proteins, leading to intravascular erythrocyte lysis due to uncontrolled complement activation. Inhibition of fD by Danicopan resulted in decreased intravascular erythrocytes lysis, but also resulted in a substantial inhibition of AP activity,^15^ which potentially increases susceptibility for infections. Therefore, these patients are vaccinated against *N. meningitidis*, *H. influenzae* and *S. pneumoniae*.

Complement activation contributes to the defence against bacterial infections by C3b‐deposition on the bacterial surface. This facilitates phagocytosis and forms the starting point of the assembly of the MAC in the outer membrane. In order to be efficient, the speed of C3b deposition and the subsequent killing machinery should be faster than the proliferation rate of the bacterium. The aim of the AP is to accelerate C3b deposition on the bacterial surface. Therefore, absence of AP activation increases the chance for invasive bacterial infections with species that proliferate rapidly in the bloodstream.^2^, ^3^, ^4^, ^5^, ^6^, ^7^, ^8^, ^9^ Here, we show that lowered fD levels decrease AP activation speed, which points towards a novel type of immunodeficiency that is characterised by an intact but too slow complement response, which predisposes to infections with rapidly proliferating bacteria that demand a rapidly accelerating response of complement.

In this study, we show by a series of experiments that the speed of AP complement activation is considerably decreased at fD levels < 0.5 µg mL^−1^. These results are in line with a dose‐dependent increase in AP50 as measured by erythrocyte haemolysis observed in experiments performed by Biesma *et al*. and Hiemstra *et al*.^3^, ^5^ In addition, we show that the widely used AP assay based on Seelen *et al*.^16^ perfectly identifies partial fD deficiency with clinical significance. We also show that fD concentration affected complement C3 deposition on the bacterial surface in a species‐specific manner. Complement C3 deposition with fD‐deficient serum, which is entirely CP‐ and LP‐dependent because of the AP‐pathway deficiency, differed between bacterial species, with Nm having the highest C3 deposition measured. For Nm, addition of only 0.09 µg mL^−1^ fD resulted in a half maximal increase in complement C3 deposition, indicating that for this type of infection only very low fD levels are needed to offer protection. Vaccination, boosting the CP through an increased binding of IgG to the bacterial surface, completely compensates for the absence of fD.

In conclusion, we show that low fD levels (< 0.5 µg mL^−1^) result in a more indolent complement activation response, revealing a type of immunodeficiency that may become clinically overt only in the absence of sufficient antibody‐mediated CP activation and during bacterial infections that require a rapidly accelerating complement activation response.

## Methods

### Ethics statement

Tests with sera from the two siblings were performed as part of diagnostic functional testing to assess the relevance of the heterozygous fD mutation, in order to provide optimal patient care and counselling.^17^ The protocols for blood collection from healthy volunteers and the previously described patient with homozygous fD mutation were approved by the ethics committee of the Radboudumc, Nijmegen.^6^ Patients and their parents gave oral informed consent for blood sample collection. Healthy volunteers gave written informed consent. All experiments were carried out in accordance with local guidelines and regulations and comply with the Declaration of Helsinki and the Good Clinical Practice guidelines.

### Exome sequencing procedure

Analysis of 386 primary immunodeficiency genes using clinical exome sequencing was done as described previously.^18^ For the gene panel analysis, a bioinformatic in silico filter was applied to select for variants affecting 386 PID genes.^19^

### Complement factor D ELISA

A Nunc MaxiSorp ELISA plate was coated with 1 µg mL^−1^ mice anti‐human fD (clone D10/4, Thermo Fisher Scientific, Waltham, MA, USA) in coating buffer (0.05 m carbonate buffer, buffer pH 9.6) and incubated overnight at 4°C. The plate was washed 3x with wash buffer [0.1% Tween 20 in phosphate‐buffered saline (PBS)]. The ELISA plate was blocked for 1 h with 2% Casein in PBS followed by washing 3× with wash buffer. Human fD standards (A136, CompTech, Tyler, TX, USA) (final concentration 500 ng mL^−1^ through 0.69 ng mL^−1^) and patient plasma (1:2, 1:10, 1:50, 1:150 and 1:450) were diluted in PBS + 0.2% Tween 20 + 10 mm EDTA and added to the wells, incubated for 60 min at room temperature, followed by washing 3× with wash buffer. Biotin‐conjugated mouse anti‐human fD (clone J‐8/1; Thermo Fisher Scientific) was diluted to 0.5 µg mL^−1^ in PBS + 0.2% Tween 20, incubated for 60 min at room temperature followed by washing 3× with wash buffer. Streptavidin‐Peroxidase (GE Healthcare, Freiburg, Germany) was diluted 1000‐fold diluted in PBS + 0.2% Tween 20, incubated for 60 min at room temperature followed by washing 3× with wash buffer. o‐Phenylenediamine (0.4 mg mL^−1^) + 0.1% H_2_O_2_ in 50 mm phosphate‐citrate buffer pH 5.0 was added to the ELISA plate, and the reaction was stopped after 5 min by adding 1.2 m H_2_SO_4_. The absorbance was measured at 492 nm and at 690 nm to control for background signal. Normal reference values for fD are 0.96–2.13 µg mL^−1^.

### Classical and alternative pathway activity assay

CP and AP activity analyses were based on Fredrikson *et al*.^20^ and Seelen *et al*.^16^. For the CP analysis, a Nunc MaxiSorp ELISA plate was coated with 120 µL 200 µg mL^−1^ human IgG (0.05 m carbonate buffer, pH 9.6) and incubated overnight at 4°C. For the AP analysis, a Nunc MaxiSorp ELISA plate was coated with 8 µg mL^−1^ *Salmonella typhosa* LPS (Sigma‐Aldrich, St. Louis, MO, USA) (AP) in 120 µL PBS by drying for 2 days at 37°C. The ELISA plate was blocked for 1 h with 1% gelatin in Tris‐buffered saline (TBS). Plates were washed 2× with wash buffer (0.01% Tween 20 in TBS) using a microplate washer (Tecan, Durham, NC, USA). Serum was diluted 40× (CP) in dilution buffer (10 mm Tris, 84 mm NaCl, 0.5 mm MgCl_2_, 2 mm CaCl_2_, 0.1% gelatin) or 10× (AP) in dilution buffer (10 mm Tris, 140 mm NaCl, 5 mm MgCl_2_, 10 mm EGTA, 0.1% gelatin), added to the wells in duplicate, and incubated for 60 min (CP) or 75 min (AP) at 37°C. For time‐course experiments, the incubation time was 30, 45, 60 or 75 min for both AP and CP analyses. Plates were washed 2× with wash buffer. Mouse aE11 monoclonal anti‐C5b‐9 (sc‐58935; Santa‐Cruz Biotechnologies, Santa Cruz, CA, USA) was diluted 1000‐fold in wash buffer, added to the wells and incubated 1 h at room temperature while shaking at 600 rpm, followed by washing 2× with wash buffer. Alkaline phosphatase‐labelled goat anti‐mouse Ig (A7434; Sigma) was diluted 2000‐fold in wash buffer, added to the wells and incubated 1 h at room temperature while shaking at 600 rpm, followed by washing 2× with wash buffer. P‐nitrophenylposphate (1 mg mL^−1^) in substrate buffer (1 m diethanolamine, 0.5 mm MgCl_2_ pH 9.8) was added, and the reaction was stopped after 15–20 min by adding 2 m NaOH. The absorbance was measured at 405 nm using a 96‐well plate reader (Tecan). Pooled human serum was used as control. Normal reference values for AP are 67–133%, and normal reference values for CP are 67–149%.

Wieslab Complement System Alternative Pathway ELISA (SVAR Life Sciences, Malmö, Sweden) was used following the manufacturer's instructions.

### Bacterial strains and growth conditions

The non‐typeable *H. influenzae* (NTHi) strain 11P6H was isolated from an adult during an acute exacerbation of COPD,^21^ and NTHi strain 86‐028NP was isolated from a child with chronic otitis media.^22^ *Streptococcus pneumoniae* strain PBCN0460 (serotype 18C) was isolated from blood of adults with bacteraemia.^23^ *S. pneumoniae* strain TIGR4 (serotype 4) is a widely used virulent laboratory isolate,^24^ and *N. meningitidis* strain H44/76 (serogroup B) and C11 (serogroup C) are sequenced laboratory isolates.^25^, ^26^ NTHi strains were cultured overnight at 37°C + 5% CO_2_ on brain heart infusion (BHI) agar plates supplemented with 1 µg mL^−1^ hemin (Sigma‐Aldrich) and 2 µg mL^−1^ ß‐nicotinamide adenine dinucleotide (Merck, Darmstadt, Germany) (sBHI). NTHi strains were subsequently grown in sBHI broth to an optical density at 620 nm (OD_620_) of 0.6 while shaking at 250 rpm and 1 mL aliquots with 16% glycerol were stored at −80°C for further experiments. *S. pneumoniae* strains were cultured overnight at 37°C + 5% CO_2_ on Columbia blood agar plates (Becton Dickinson, BD Bioscience, Franklin Lakes, NJ, USA). *S. pneumoniae* strains were subsequently grown in Todd‐Hewitt broth supplemented with 5 g L^−1^ yeast extract (THY) at 37°C and 5% CO_2_ to an OD_620_ of 0.3 and 1 mL aliquots with 16% glycerol were stored at −80°C for further experiments. *N. meningitidis* was cultured overnight at 37°C + 5% CO_2_ on CG agar plates (BD Biosciences, San Jose, CA, USA). *N. meningitidis* strains were subsequently grown in GC broth at 37°C to an OD_620_ of 0.3 while shaking at 250 rpm and were used directly in experiments. The number of colony‐forming units (CFU) per mL was determined by plating serial 10‐fold dilutions on agar plates.

### Flow cytometric analysis

Frozen bacteria stocks were thawed, washed once with HBSS + Ca^2+^/Mg^2+^ + 0.1% gelatin (HBSS3+) and diluted to an OD_620_ of 0.1 with HBSS3+. A volume of 25 µL of bacteria were mixed with 25 µL 10% human serum (for C3 binding) or 10% heat‐inactivated (HI) (20 min 56°C) serum (for IgG binding) in HBSS3+ with or without reconstitution of fD (A136; CompTech) reconstitution and incubated 0, 5, 15 or 30 min at 37°C. Complement activation was stopped by adding 5 mm EDTA and cooling on ice. Bacteria were pelleted by centrifugation at 3200 *g* and fixed for 20 min in 2% paraformaldehyde in PBS at room temperature. Surface‐bound complement C3 was detected with 1:500‐diluted FITC‐labelled polyclonal goat anti‐human C3 (MP Biomedicals, Solon OH, USA). Surface‐bound IgG was detected with 1:500‐diluted Fcγ fragment‐specific PE‐labelled AffiniPure goat anti‐human IgG (Jackson ImmunoResearch, West Grove, PA, USA). Surface binding of C3 or IgG was determined by flow cytometry using a FACS LSR II instrument (BD Biosciences) and expressed in mean fluorescence intensity in arbitrary units. Data were analysed by using FlowJo version 10.4.1. (FlowJo LLC, Ashland, OR).

### Serum killing assay

Frozen NTHi stocks were thawed, washed once with PBS, diluted to an OD_620_ = 0.1 in PBS and diluted 2500‐fold in HBSS3+ to obtain a concentration of ~200 000 CFU mL^−1^. *N. meningitidis* was grown fresh in GC broth at 37°C and 5% CO_2_ to an OD620 of 0.23, washed once with PBS and diluted 1000‐fold in HBSS3+ to obtain a concentration of ~200 000 CFU mL^−1^. A volume of 25 µL of bacteria were mixed with 25 µL 10% serum with or without reconstitution of fD or with 10% HI serum and incubated for 30 min (*N. meningitidis*) or 60 min (NTHi) at 37°C. Samples were diluted 10‐ and 100‐fold with PBS, and three droplets of 20 µL of the undiluted, 10 and 100‐fold diluted bacteria were plated on agar plates and grown overnight at 37°C and 5% CO_2_. Survival was determined by dividing the CFU counts in NHS by the CFU count in HI‐serum after 30 min (*N. meningitidis*) or 60 min (NTHi) incubation and presented as percentage.

### Statistical analysis

Statistical analyses were performed with GraphPad Prism (Graphpad, San Diego, CA, USA) version 5.03 for Windows. Differences were considered significant at *P*‐value < 0.05. The specific statistical tests that were used for the various experiments are specified in the figure captions.

## Conflict of interest

The authors declare no conflict of interest.

## Author contributions

**Jeroen D Langereis:** Conceptualization, Methodology, Investigation, Formal Analysis, Writing – Original Draft, Writing – Review & Editing. **Renate G van der Molen:** Resources, Conceptualization, Methodology, Writing – Review & Editing. **Marien I de Jonge:** Conceptualization, Methodology, Writing – Review & Editing. **Corrie de Kat Angelino:** Investigation; Writing‐review & editing. **Stefanie Henriet:** Investigation; Methodology; Writing‐review & editing. **Irma Joosten:** Supervision; Writing‐review & editing. **Annet Simons:** Investigation; Writing‐review & editing. **Janneke Schuurs‐Hoeijmakers:** Investigation; Writing‐review & editing. **Marcel van Deuren:** Investigation; Methodology; Writing‐review & editing. **Koen van Aerde:** Investigation; Writing‐review & editing. **Michiel van der Flier:** Conceptualization, Resources, Writing – Review & Editing.
